# Global Population Structure of the Genes Encoding the Malaria Vaccine Candidate, *Plasmodium vivax* Apical Membrane Antigen 1 (*Pv*AMA1)

**DOI:** 10.1371/journal.pntd.0002506

**Published:** 2013-10-31

**Authors:** Alicia Arnott, Ivo Mueller, Paul A. Ramsland, Peter M. Siba, John C. Reeder, Alyssa E. Barry

**Affiliations:** 1 Centre for Biomedical Research, Burnet Institute, Melbourne, Australia; 2 Barcelona Centre for International Health Research, Barcelona, Spain; 3 Division of Infection and Immunity, Walter and Eliza Hall Institute of Medical Research, Melbourne, Australia; 4 Department of Medical Biology, University of Melbourne, Parkville, Australia; 5 Department of Immunology, Monash University, Melbourne, Australia; 6 Department of Surgery Austin Health, University of Melbourne, Heidelberg, Australia; 7 School of Biomedical Sciences, CHIRI Biosciences, Faculty of Health Sciences, Curtin University, Perth, Australia; 8 Papua New Guinea Institute for Medical Research, Goroka, Papua New Guinea; 9 Centre for Population Health, Burnet Institute, Melbourne, Australia; 10 Department of Epidemiology and Preventative Medicine, Monash University, Melbourne, Australia; Barcelona Centre for International Health Research (CRESIB) and Institució Catalana de Recerca i Estudis Avançats (ICREA), Spain

## Abstract

**Background:**

The *Plasmodium vivax* Apical Membrane Antigen 1 (*Pv*AMA1) is a promising malaria vaccine candidate, however it remains unclear which regions are naturally targeted by host immunity and whether its high genetic diversity will preclude coverage by a monovalent vaccine. To assess its feasibility as a vaccine candidate, we investigated the global population structure of *Pv*AMA1.

**Methodology and Principal Findings:**

New sequences from Papua New Guinea (PNG, n = 102) were analysed together with published sequences from Thailand (n = 158), India (n = 8), Sri Lanka (n = 23), Venezuela (n = 74) and a collection of isolates from disparate geographic locations (n = 8). A total of 92 single nucleotide polymorphisms (SNPs) were identified including 22 synonymous SNPs and 70 non-synonymous (NS) SNPs. Polymorphisms and signatures of balancing (positive Tajima's D and low *F_ST_* values) selection were predominantly clustered in domain I, suggesting it is a dominant target of protective immune responses. To estimate global antigenic diversity, haplotypes comprised of (i) non-singleton (n = 40) and (ii) common (≥10% minor allele frequency, n = 23) polymorphic amino acid sites were then analysed revealing a total of 219 and 210 distinct haplotypes, respectively. Although highly diverse, the 210 haplotypes comprised of only common polymorphisms were grouped into eleven clusters, however substantial geographic differentiation was observed, and this may have implications for the efficacy of *Pv*AMA1 vaccines in different malaria-endemic areas. The PNG haplotypes form a distinct group of clusters not found in any other geographic region. Vaccine haplotypes were rare and geographically restricted, suggesting potentially poor efficacy of candidate *Pv*AMA1 vaccines.

**Conclusions:**

It may be possible to cover the existing global *Pv*AMA1 diversity by selection of diverse alleles based on these analyses however it will be important to first define the relationships between the genetic and antigenic diversity of this molecule.

## Author Summary

Traditionally misclassified as benign and neglected as a research priority, it is now understood that *P. vivax* is an increasingly important cause of human malaria. This important human pathogen poses an enormous obstacle to malaria control and elimination efforts due its broad geographic distribution, ability to cause recurring episodes of malaria after long periods of inactivity and extreme biodiversity. Vaccines are an essential component of global malaria control and elimination campaigns but the diversity of malaria antigens is thought to be a major cause of vaccine failure. Furthermore, at present the majority of current vaccine research is directed toward *P. falciparum.* The aims of this study were to investigate the global diversity of the *P. vivax* vaccine candidate, Apical Membrane Antigen 1 (*Pv*AMA1), to determine the feasibility of designing a globally effective *Pv*AMA1 vaccine and to determine which region of *Pv*AMA1 is targeted by host immune responses, in order to identify the most promising vaccine candidates. We report that *Pv*AMA1 diversity is extremely high, and that *Pv*AMA1 domain I is a dominant target of host immune responses. These analyses of *Pv*AMA1 diversity from several geographic regions provide a framework to guide development of a broadly efficacious *P. vivax* vaccine.

## Introduction

More than 2.5 billion people live at risk of *Plasmodium vivax* infection [1]. Although it has been traditionally misclassified as a ‘benign’ infection, *P. vivax* is now known to result in serious illness and death [2], [3]. Research into *P. vivax* has been neglected and much remains unknown regarding the biology, pathogenesis and epidemiology of this parasite [4]. As a result, *P. vivax* remains a substantial obstacle to malaria control and elimination programs, as demonstrated in countries that have made progress in reducing the burden of the other major human malaria parasite, *Plasmodium falciparum* [5]. Worldwide, the burden of *P. vivax* has been increasing [6], [7], [8] in the context of a substantial decrease in *P. falciparum* cases [9], but research on novel *P. vivax* drug and vaccine targets currently lags far behind that of *P. falciparum*. To date only two *P. vivax* candidate vaccines have reached Phase I clinical trials, as compared to 23 *P. falciparum* vaccine candidates, several of which have progressed to Phase II and III clinical trials [10].

The Apical Membrane Antigen 1 (AMA1) is found in all *Plasmodium* spp. and parasites of the Apicomplexa phylum [11], [12], and is a promising vaccine candidate for both *P. falciparum* and *P. vivax* [11], [13]. AMA1 is an 83 kDa type 1 integral membrane protein consisting of a signal sequence, a cysteine-rich ectodomain, a conserved cytoplasmic region and a transmembrane region. Disulfide bonds between cysteine residues of the AMA1 ectodomain promote separation of the ectodomain into three distinct domains (DI–DIII) [14]. From experiments performed using *P. falciparum* and *Toxoplasma gondii*, it is now known that invasion of host cells involves the moving junction (MJ) complex, which enables internalization of the parasite into the host cell [15], [16]. The MJ contains AMA1 and unique Apicomplexa proteins called rhoptry neck (RON) proteins. In particular, the RON2 protein has been shown to interact directly with AMA1. Blocking this interaction inhibits parasite invasion [17]. In the Apicomplexan parasite *T. gondii*, conditional AMA1 null parasites could not invade host cells [18]. Similarly, AMA1 has been shown to be essential for *P. falciparum* invasion of host cells [12], [19], [20].

During invasion, the bulk of the AMA1 ectodomain is shed, leaving only the 22 kDa cytoplasmic tail which is carried into the host cell [21]. A consequence of this shedding is evasion of anti-AMA1 immune responses [19]. Immuno-epidemiological studies have demonstrated that the ectodomains of both *P. falciparum* and *P. vivax* AMA1 are highly immunogenic [11], [22], [23]. In human populations that are naturally exposed to malaria, AMA1 antibody levels are higher than those of other blood-stage antigens [11], [22] and these have been shown to inhibit *P. falciparum* invasion of host cells [24], [25], [26]. As AMA1 is essential for invasion of both host erythrocytes by merozoites and sporozoite invasion of hepatocytes, a vaccine that induces antibodies to AMA1 may be effective at two distinct life-cycle stages, thus increasing the likelihood of protection against malaria [12].

Domains I (DI) and II (DII) of AMA1 share a common core topology, namely a pair of closely associated PAN (*P*lasminogen, *A*pple, *N*ematode) domains [12]. Loops of DI and DII extend from the PAN domains to surround and contribute to the base and sides of a long hydrophobic cleft, created by the interaction of DI and DII on the AMA1 surface [12], [27]. The cleft is a highly conserved ligand-binding site [17], [28]. It is therefore hypothesised that the highly polymorphic loop structures provide conformational masking to protect the binding site from host antibody responses [28]. This is supported by the fact that many of the most polymorphic sites are located on the loops of DI, at one end of the cleft [27], [28]. Accordingly, for *P. falciparum*, the majority of the genetic diversity occurs in DI [29], [30], [31], however it has been reported that both DI and DII are highly polymorphic for *P. vivax* [29], [32]. Analyses of full-length *P. vivax* AMA1 (*Pvama1*) ectodomain sequences from Venezuela suggest that balancing (immune) selection is predominant in DI [29]. Evidence of balancing selection on DI has also been reported in other parasite populations, however many of these studies investigated sequence diversity only within DI and not the entire ectodomain [33], [34], [35]. Evidence of diversifying selection on DII was observed among full-length *Pvama1* nucleotide sequences from Sri Lanka, suggesting that regions of DII may also be targeted by protective immune responses [32]. Furthermore, serological studies have shown DII to be the most immunogenic of the three domains [22]. Therefore, although the hydrophobic cleft found in DI is clearly the target of host immune responses, it is likely that antibodies also recognize epitopes in other regions of the *Pv*AMA1 ectodomain. It remains unclear which domain is predominantly targeted by protective host immune responses and therefore which domain(s) should be incorporated into a *Pv*AMA1-based vaccine.

To design an effective *Pv*AMA1-based malaria vaccine, it is important not only to identify the region representing the strongest natural immune target, but also to include alleles that induce antibodies with broad reactivity to cover the antigenic diversity of the global *P. vivax* population [36]. It is therefore also important to understand the population structure of vaccine candidate antigens, to predict key polymorphisms that contribute to antigenic diversity [12], [37], [38] and to investigate the geographical distribution of antigen diversity. Population genetic studies are thus needed to guide informed vaccine design [39], [40].

Presented here is the most comprehensive study of *Pvama1* genetic diversity undertaken to date. The aims of this study were to investigate the population structure of *Pvama1* in two highly malaria endemic regions of Papua New Guinea (PNG), and to compare the data to published *Pvama1* sequences from other parasite populations to identify polymorphisms likely to be vaccine-relevant. The results of this study have important implications for the design of a vaccine incorporating *Pv*AMA1, with respect to selection of the optimal target region of the protein, and the haplotypes required to cover diversity.

## Materials and Methods

### Study sites and *Plasmodium vivax* isolates

Within PNG, the four main malaria species that infect humans (*P. falciparum*, *P. vivax*, *P. ovale* and *P. malariae*) are endemic, and mixed species infections are common [7], [41]. The PNG provinces of Madang and East Sepik are areas of intense perennial malaria transmission and have long been the focus of malaria research and control efforts in PNG. To capture a broad cross-section of the circulating parasite population, venous blood samples were collected from volunteers of all ages in cross-sectional malaria surveys conducted in the catchment areas of Mugil, Malala and Utu in Madang Province and the Wosera district in the East Sepik Province. Because studies using microsatellite markers have indicated limited geographic population structure of *P. vivax* in PNG [42], samples collected from the three different catchments in Madang Province were combined to form a single population for comparison to that from the more distant Wosera, East Sepik parasite population. Nevertheless, data for the four different catchments (Mugil, Malala, Utu and Wosera) were also analysed separately to investigate possible differences in *Pv*AMA1 between catchments. The *P. vivax* isolates and the study sites are described in more detail elsewhere [43], [44]. A total of 102 monoclonal *P. vivax* isolates were identified by genotyping *P. vivax* positive samples at the highly polymorphic loci MS16 and *msp1*F3 according to published methods [45].

### Ethics statement

Samples archived in a biobank at the PNG Institute of Medical Research were used in the study. The original study in which samples were collected was explained in detail to the parents through both individual and community awareness meetings. On the basis of these explanations, volunteers were invited to participate in the study. During enrolment, adult volunteers or the legal guardians of child volunteers were asked to provide oral informed consent to participate in the study as this was the ethical requirement for this particular study, as approved by the local Institutional Review Board (see details below). Oral consent to participate in the study and for samples to be used in further research was documented in a database. Enrolment in the study was possible only if consent was given. All consenting members of selected populations were eligible for enrolment into the community surveys. People with concurrent or chronic illness that might impede their participation in the surveys were excluded. Ethical approval to conduct this study was granted by the PNG Institute of Medical Research Institutional Review Board (No. 11-05), the Medical Research Advisory Committee of PNG (No. 11-06), the Alfred Hospital Research and Ethics Unit (No. 420-10) and the Walter and Eliza Hall Institute Human Research Ethics Committee (No. 11-12).

### *Pvama1* PCR and sequencing

Using a previously described nested PCR approach [29], [46], nucleotides 1-1524 of the 1686 bp *Pvama1* coding sequence were amplified and sequenced. This region encompasses the signal sequence and the complete ectodomain (DI to DIII) [29]. Sequencing reactions were performed by a contract sequencing facility using the ABI BigDye Terminator Cycle Sequencing kit on an ABI 3730XL automatic DNA Analyser (Macrogen, Seoul, Korea).

### Sequence analysis

Raw sequence data was edited and assembled into contigs using Sequencher version 5.0 [47]. Single nucleotide polymorphisms (SNPs) were identified by comparing the consensus sequences for each isolate to the reference *Pvama1* sequence, *Sal-1* (GenBank accession number AF063138; Table S1). Individual SNPs were validated if they were present in at least one other isolate. Rare SNPs (frequency = 1) were confirmed by sequencing a second independent PCR product.

To investigate the worldwide population structure of *Pvama1*, 263 previously published full-length ectodomain sequences were retrieved from GenBank (Table S1). These *Pvama1* sequences represented the parasite populations of seven distinct locations, including one in Venezuela sampled at two different time points (Amazon Basin 1996 and 1997, n = 73) [29], two regions of Thailand with one region sampled at two different time points (Chanthaburi and Tak 1996 and 2007, n = 158) [48], India (Rajasthan, n = 8) [49], Sri Lanka (Kataragama-Colombo, n = 23) [32] and South Korea (n = 1) (Table S1). A fourth Thai population was excluded, because diversity within this population was found to be extremely low (4 haplotypes among 73 isolates) and is reportedly due to rapid clonal population expansion from a small number of founder isolates [48]. The *Pvama1* sequences of six primate-adapted *P. vivax* isolates used for vaccine research (*Chesson I*, *Belem*, *India VII*, *Indonesia XIX*, *Palo Alto*, *North Korea*; [50]) and *Sal-1*, were also obtained from GenBank and included in the analyses (Table S1).

Alignment and phylogenetic analysis of *Pvama1* sequences was performed using MEGA version 5.0 [51]. A neighbor-joining tree was constructed using DNA sequences encoding the full ectodomain and the *p*-distance nucleotide substitution model with 1000 bootstrap replicates, to investigate the relatedness between *Pvama1* alleles circulating in the East Sepik and Madang provinces of PNG.

Allele frequencies were calculated using CONVERT version 1.31 [52]. The following analyses were performed using DnaSP version 5.0 [53]: several diversity statistics were calculated for the worldwide dataset and each individual population including the total number of polymorphisms (*S*), the number of synonymous (*SP*) and non-synonymous (*NS*) polymorphisms, nucleotide diversity (*Π*; Pi), the number of haplotypes (*h*) and haplotype diversity (*Hd*). Haplotype diversity is analogous to the expected heterozygosity and is calculated as follows:
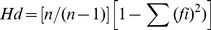
where n is the sample size and *f* is the frequency of the *i* ^th^ allele [54].

For the entire dataset, nucleotide diversity was calculated across the length of the gene using a sliding window approach, with a window size of 100 and step size of 3. To identify significant departures from neutral evolution the Tajima's D test statistic [55] was also calculated for each population with a sample size >30 using a sliding window approach with a window size of 100 and a step size of 3. Balancing selection (measured using the Tajima's D test) maintains alleles at balanced frequencies within populations, resulting in very low levels of local interpopulation differentiation (*F_ST_*) at selected loci [39].

To focus the analysis on polymorphisms affecting protein structure and therefore with the potential to influence antigenic diversity, haplotypes were constructed using all of the non-singleton amino acid polymorphisms. Amino acid polymorphisms were analysed rather than DNA due to the presence of complex codons with multiple non-synonymous polymorphisms. Two haplotype datasets were prepared: (i) all 40 non-singleton amino acid polymorphisms, and (ii) a minimal haplotype comprised of 23 amino acid polymorphisms ranking highly for relevance to vaccine design. The ranking criteria used to determine relevant polymorphisms included a minor allele frequency (MAF)≥10% which excluded rare polymorphisms [56] and where possible, evidence of balancing selection including a significant Tajima's D value and low levels of interpopulation differentiation, measured by determining pairwise *F_ST_* values for each polymorphic site [57], [58]. Low *F*_ST_ (≤0.15) suggests maintenance of balanced allele frequencies as a result of immune selection; whereas the frequency of polymorphisms not under selection is likely to change at random and result in higher *F*_ST_ values (≥0.25). This analysis could only be performed for countries where parasite populations were available from more than one geographic region (i.e. Thailand and PNG); other populations could not be included because geographic population structure between continents may obscure balancing selection [39]. The software GENEPOP version 4.1.1 [59], [60], with all parameters set at default values, was used to calculate *F_ST_*.

To identify groups of closely-related haplotypes likely to be “antigenically similar”, and their geographic distribution, the haplotypes were analysed using the Bayesian clustering algorithm implemented in Structure version 2.3.2 software [61], [62]. This Bayesian clustering algorithm groups related haplotypes into a user-defined number of clusters (*K*) on the basis of shared allele frequencies. Each haplotype is assigned a membership coefficient (*Q*) to each of the clusters, with *Q*<75% indicating an admixed haplotype likely to have arisen by recombination between genetically distinct sequences. Replicate runs of Structure were performed using a burn-in period of 10,000 iterations, followed by 10,000 Markov chain Monte Carlo (MCMC) iterations from which estimates of population numbers were obtained. All runs were based on the admixture model, with no prior population information [61]. Twenty replicate runs were performed for *K* values of 1 to 30. To predict the optimal value of K, the posterior probability of the data (Ln*P*[D]) [52] and its standard deviation were plotted as well as an ad hoc statistic based on the second order rate of change of *K*, *ΔK*, according to the method of Evanno *et al*., (2005) [63]. Geographic population structure was investigated by visually assessing whether the clusters were distributed randomly (no geographic structure) or among locations (geographic structure).

To investigate more complex relationships among haplotypes and to infer patterns of recombination amongst haplotypes and haplotype clusters, a network analysis was performed for the unique haplotypes by using *Phylogenetic Network* software version 4.6.1.1 together with the add-ons *DNA Alignment* and *Network Publisher* (fluxus-engineering.com). The network analysis was based on the Median Joining algorithm [64].

### Structural modelling of *Pv*AMA1

Homology models of *Pv*AMA1 were prepared with the Build Model protocol that uses the Modeller algorithm [65] of the Discovery Studio suite, version 3.0 (Accelrys, San Diego, CA). Using this approach, an atomic model of *Pv*AMA1 was generated from a chimeric template and sequence alignment. The chimeric template was generated using overlays of the *P. vivax* (Protein Data Bank ID: 1W81) and *P. falciparum* (Protein Data Bank ID: 1Z40) AMA1 crystal structures, and grafting *Pf*AMA1 loop residues onto the *Pv*AMA1 core. The grafted residues were as follows: *Pf*AMA1 residues 260 to 263 (numbering relative to the *P. falciparum* reference strain *3D7* AMA1 sequence) which corresponded to *Pv*AMA1 residues 205 to 208 (numbering relative to the *P. vivax* reference strain *Sal-1* AMA1 sequence); *Pf*AMA1 residues 350 to 385 which corresponded to *Pv*AMA1 residues 295 to 325; *Pf*AMA1 residues 388 and 390 which corresponded to *Pv*AMA1 residues 333 to 334. Four *Pv*AMA1 loop structures missing from both the *Pv*AMA1 and *Pf*AMA1 crystal structures were generated automatically by Modeller using only stereochemical and geometric restraints: *Pv*AMA1 residues 171 to 175 (loop 1), 209 to 218 (loop 2), 331 and 332 (loop 3), 400 to 412 (loop 4). The *P. vivax Sal-1* reference sequence (GenBank accession number AF063138) was aligned against the chimeric template sequence in order to generate the *Pv*AMA1 model. Optimisation of template-based models was achieved by iterative cycles of conjugate-gradient minimisation against a probability density function (PDF) that included spatial restraints derived from the template and residue specific properties [65]. Five models were generated and the optimised model with the lowest final PDF energy was used for structural analysis and figure preparation using Discovery Studio, version 3.1.

### Accession numbers

The 102 *Pvama1* sequences generated in this study were deposited in GenBank under the accession numbers KC702402–KC702503.

## Results

### Polymorphism and genetic diversity of the genes encoding *Pv*AMA1 in two parasite populations of Papua New Guinea

Complete *Pvama1* sequences encoding the ectodomain (nucleotides 1-1524 relative to *Sal-1*, [GenBank accession number: AF063138]) were obtained for 102 monoclonal *P. vivax* isolates from PNG, which included 41 sequences from the East Sepik Province (ES) and 61 from Madang Province (Mad). Multiple DNA sequence alignments revealed a total of 46 SNPs, including 7 synonymous (SP: ES = 9, Mad = 12) and 39 non-synonymous (NS: ES = 30, Mad = 31; Table 1).

**Table 1:** Estimates of genetic diversity for *Pvama1* in nine worldwide populations.

Population	n	S	Π (×10^−3^)	NS	SP	NS SNP haplotypes^a^		Amino acid haplotypes^b^	Minimal amino acid haplotypes^c^
						h	Hd	h	h
**PNG**	**102**	**44**	**7.9**	**39**	**7**	**87**	0.99	**87**	**82**
East Sepik	41	37	7.5	30	9	38	0.99	38	36
Madang	61	40	8.0	31	12	52	0.99	52	51
**Thailand**	**158**	**57**	**8.9**	**42**	**14**	**90**	0.98	**91**	**89**
Tak 1996	58	49	8.4	34	13	52	0.99	53	52
Tak 2007	44	48	8.8	35	10	31	0.98	30	31
Chanthaburi	56	47	8.9	35	11	25	0.89	25	25
**Venezuela**	**73**	**29**	**6.5**	**23**	**6**	**17**	0.90	**17**	**17**
1996	28	25	5.6	18	8	6	0.79	6	6
1997	45	29	6.4	21	8	16	0.91	16	16
**India**	**8**	**27**	**7.0**	**20**	**5**	**6**	0.92	**6**	**6**
**Sri Lanka**	**23**	**34**	**6.8**	**27**	**5**	**15**	0.94	**15**	**15**
**Reference strains**	**7**	**44**	**10**	**35**	**11**	**6**	0.95	**6**	**6**
**Total** d	**372**	**93**	**9.3**	**70**	**22**	**218**	**0.99**	**219**	**210**

n = number of samples; S = number of polymorphic sites; Π = nucleotide diversity; NS = number of non-synonymous polymorphisms; SP = number of synonymous polymorphisms; h = number of haplotypes; Hd = haplotype diversity. Totals for each country and population of *Pvama1* sequences investigated are shown in bold.

a = number and diversity of haplotypes constructed using all observed non-singleton, non-synonymous SNPs.

b = number and diversity of haplotypes constructed using the 40 non-singleton, non-synonymous amino acid polymorphisms.

c = number and diversity of haplotypes constructed using the 23 non-singleton, non-synonymous amino acid polymorphisms.

d = totals include the reference strains and the single South Korean sequence which is absent from the table body as the population analyses performed could not be done on a single sequence.

Amongst the 102 PNG *Pvama1* sequences obtained, a total of 87 NS-SNP haplotypes were identified (ES = 38, Mad = 52), 84 of which were restricted to one of the two populations (ES = 35, Mad = 49) (Table 1). Haplotype diversity was close to the highest possible level (*Hd*>0.99; Table 1). Despite the majority of haplotypes being unique to each of the PNG populations, *Pvama1* sequences from the ES and Mad populations did not form distinct clades by phylogenetic analyses (Figure S1).

### Polymorphism and genetic diversity of the genes encoding *Pv*AMA1 in eight worldwide parasite populations

The *Pvama1* sequence data obtained from PNG (n = 102) was then compared to previously published sequences from three populations in Thailand (Chanthaburi and Tak 1996 and 2007; n = 158), two from Venezuela (Amazon Basin 1996 and 1997; n = 73) and one each from India (n = 8) and Sri Lanka (n = 23). Also included in the analyses was a single *P. vivax* isolate from South Korea (n = 1) and seven geographically diverse reference isolates (n = 7). Hereafter, when referring to sequence datasets used in this paper, ‘global’ refers to a combined dataset consisting of the PNG *Pvama1* sequences obtained in this study and the published *Pvama1* sequences indicated above. A total of 92 SNPs were identified amongst the 372 global and reference *Pvama1* sequences, including 22 SP SNPs and 70 NS SNPs (Table 1).

**Figure 1:**
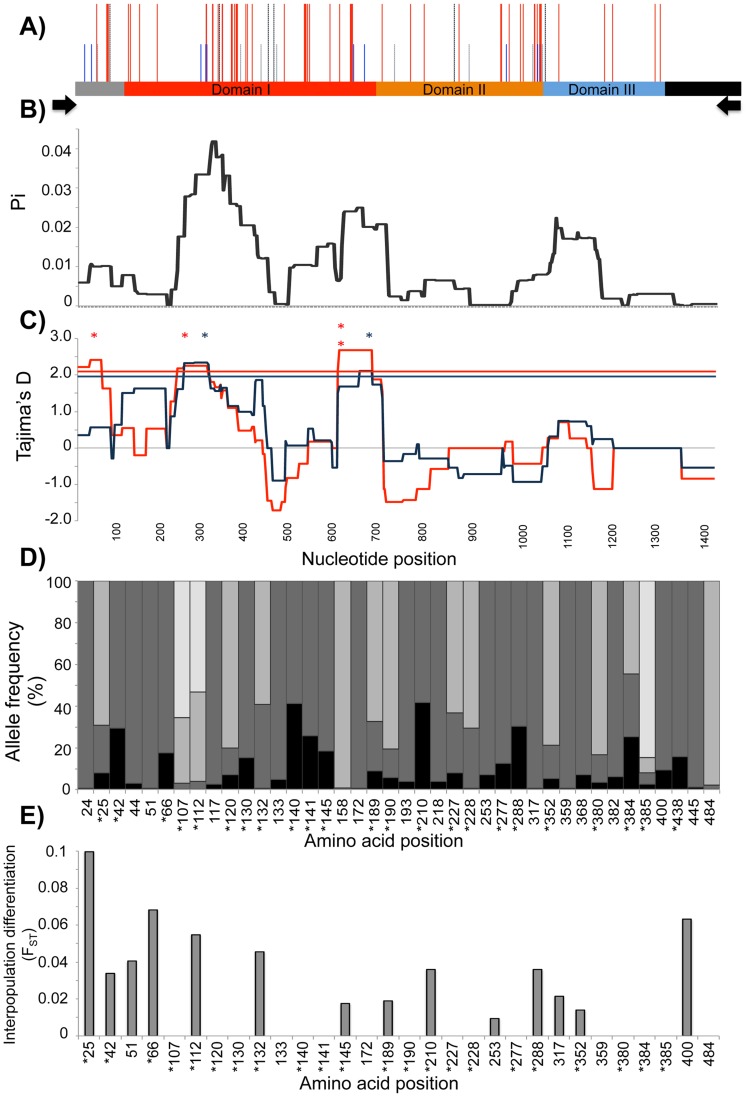
Population genetics of the genes encoding the *Pv*AMA1 ectodomain. A) Polymorphism. Schematic of the *Pvama1* region analysed indicating the locations of all nonsynonymous (NS, red lines), synonymous (SP, blue lines) and NS and SP singleton (black and grey lines, respectively) SNPs. Black arrows indicate the positions of PCR primers. B) Diversity. Sliding window analysis showing nucleotide diversity (Π; Pi) values in *Pvama1* for the 372 sequences analysed. A window size of 100 bp and a step size of 3 bp were used. C) Natural selection. Sliding window calculation of Tajima's D statistic was performed for the two PNG populations (blue = Madang, n = 61; red = East Sepik, n = 41). A window size of 100 and a step size of 3 were used. Horizontal dashed lines indicates the significance threshold of p = 0.05; A single asterisk indicates significance values for which p<0.05; and double asterisk indicates p<0.01. D) Amino acid allele frequencies. The frequencies of 40 non-singleton NS amino acid polymorphisms are indicated by the proportion of each bar shaded. Polymorphisms with minor allele frequencies (MAF)≥10% as indicated by an asterisk, were used for further analyses. E) Interpopulation differentiation. Pairwise *F*_ST_ values were calculated for the two PNG populations at each of the 40 non-singleton NS amino acid polymorphisms. Those with a MAF≥10% are indicated by an asterisk.

The majority of NS SNPs (72.5%) clustered within domain I, demonstrated by a peak in nucleotide diversity (Π) for this region (Figures 1A and 1B; Figure S2). Although a cluster of SNPs was also observed at the junction between domains II and III, values of Π in this region were lower than that observed for DI and DII due to the fact that these were lower frequency polymorphisms (Frequency = 0.11–0.23; Figure 1B). Even with singletons removed, haplotype diversity was extremely high for the global dataset (*Hd* = 0.99), with a total of 218 NS-SNP haplotypes identified (Table 1). In all populations, the genetic diversity of *Pvama1* was extremely high with predominantly NS SNPs, and extremely high Π and *Hd* values (Table 1). Diversity was highest in PNG and Thailand and lowest in Venezuela (Table 1).

### Signatures of balancing selection in the genes encoding *Pv*AMA1

To identify the predominant target(s) of balancing selection, Tajima's D values across the region encoding the *Pv*AMA1 ectodomain were individually determined for each natural population using a sliding window approach (Figure 1C, Figure S3). Significant positive values of Tajima's D indicate balancing selection, whereas negative values indicate purifying or directional selection. Although positive values of Tajima's D were observed across the length of the entire ectodomain, highly significant (p<0.01) positive values were consistently observed within DI of the PNG, Thai and Venezuelan populations (Figures 1C, and S3), which suggests this domain is a dominant target of host immune responses.

### A minimal *Pv*AMA1 haplotype relevant to malaria vaccine design

The 40 non-singleton amino acid polymorphisms identified amongst the global dataset of 372 sequences were then ranked for their potential to contribute to antigenic diversity and therefore their relevance to development of a broadly efficacious vaccine. Amino acid polymorphisms with a minor allele frequency (MAF) of ≥10% were chosen because these polymorphic sites were deemed common and thus contribute to a large proportion of the global *Pv*AMA1 diversity. If there was more than one minor allele, polymorphisms with a sum of these MAFs ≥10% were also included. Twenty-three polymorphisms met the defined criteria (Figure 1D). Additionally, the interpopulation differentiation was measured for each polymorphic site to predict sites under balancing selection. Low *F_ST_* values (<0.1) for those polymorphisms located within DI were found between the two PNG populations (Figure 1E) as well as between two Thai populations sampled in 2007 (data not shown) providing further evidence that immune selection is acting to maintain diversity at these sites [39]. Of the 23 polymorphic residues, 10 were dimorphic, 10 were trimorphic and three were tetramorphic (Table S2). Thirteen (56.5%) of the 23 variant sites were located within DI (Figure S2).

In *P. falciparum*, four clusters of polymorphic residues within AMA1 domain I, namely c1, c1L, c2 and c3, have previously been associated with antigenic escape [66]. In total, 9 of the 23 *Pv*AMA1 polymorphic amino acids mapped to the same positions as *Pf*AMA1 c1, c1L, c2 or c3 residues (Figure S2). The *Pf*AMA1 c1 cluster spans 21 residues, 7 of which are polymorphic [67], however only 4 of the 23 polymorphic *Pvama1* residues mapped to this region (Figure S2). Located within the c1 cluster, residues of the c1L subcluster are most strongly associated with *Pf*AMA1 escape from antibody-mediated inhibition [38]. Only two of the 23 ‘common’ *Pv*AMA1 polymorphisms were located within the 11-residue stretch corresponding to the *Pf*AMA1 c1L cluster (Figure S2).

**Figure 2:**
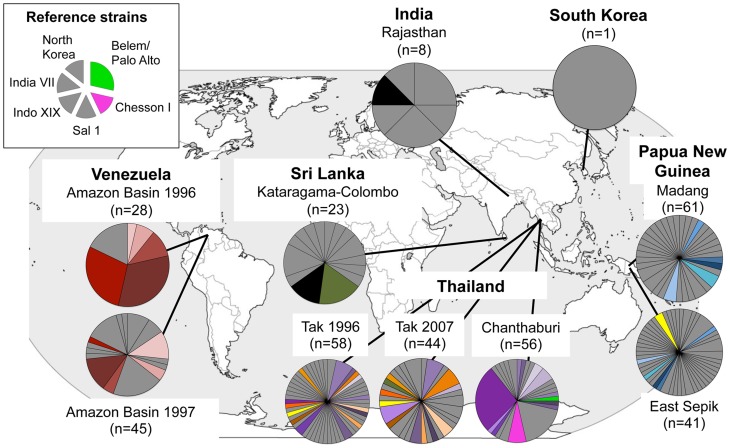
Worldwide distribution of *Pv*AMA1 haplotypes. The frequencies of 210 haplotypes based on the analysis of 23 common amino acid polymorphisms are depicted as pie charts and mapped to their geographic origin. Coloured segments indicate shared haplotypes and grey indicates haplotypes unique to one population. Only two haplotypes were identical to reference strains (*Belem/Palo Alto* and *Chesson I*), therefore haplotypes from the remaining reference strains are shown in grey. Sample size (n) and origin are indicated.

We then compared the diversity of haplotypes comprised of non-singleton polymorphisms (40-mer) and those that were common (23-mer). Despite a large decrease in the total number of polymorphisms included in the 23-mer compared to the 40-mer haplotype, there was only a marginal decrease in the number of haplotypes from 219 to 210 respectively, showing that the 23-mer haplotypes are indeed representative of a large majority of the known *Pv*AMA1 diversity (Figures 2 and S4; Table 1). Similarly, the proportion of haplotypes shared between populations was equivalent for both the 40-mer and 23-mer haplotype datasets. Twenty-five of the 219 40-mers (11.4%) were shared between populations whereas 30 of the 210 23-mers (14.2%) were shared between populations (Figures 2 and S4). However, half of the shared 23-mers were actually shared only between the three Thai populations (Figure 2) and only three (10%) were shared between populations from different countries. Notably, there were no dominant haplotypes, and the vaccine strain *Sal-1* haplotype was not identified in any of the parasite populations included in the study (Figures 2 and S4). Of the reference strain *Pv*AMA1 sequences included in the analysis, only the *Belem/Palo Alto* (which have identical haplotypes) and *Chesson I* haplotypes were detected, but these were restricted to one of the Thai populations (Chanthaburi, Figure 2).

### Polymorphic residues under balancing selection mapped exclusively to solvent-exposed surfaces mostly within *Pv*AMA1 DI and DII

A three-dimensional model of *Pv*AMA1 was generated in order understand the potential structure-function relationships of the 23 polymorphic amino acids under balancing selection. In order to determine the proximity of the polymorphic residues to the ligand binding cleft, the amino acid residues comprising the hydrophobic ligand binding cleft [27], in addition to 22 of the 23 polymorphic residues under selection, were mapped to the *Pv*AMA1 structure. One of the 23 polymorphic residues (Gln25) was located within the signal sequence and could not be mapped to the three-dimensional structure as the first 40 residues were missing from both the *Pv*AMA1 and *Pf*AMA1 crystal structures used for modelling of *Pv*AMA1.

**Figure 3:**
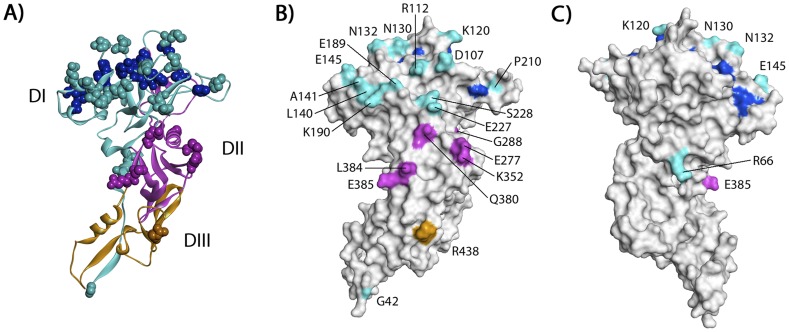
Location of 22 polymorphic *Pv*AMA1 residues predicted to be under balancing selection. A) Ribbon diagram of the PvAMA1 model showing each of the *Pv*AMA1 domains (DI in cyan, DII in magenta, and DIII in orange) and the hydrophobic ligand binding cleft (dark blue). Each of the 22 residues under selection and the 12 hydrophobic cleft residues are shown by CPK-models of their atoms (spheres) and are coloured according to location. B) Solvent-accessible surface representation of the ‘active face’ of the *Pv*AMA1 model. The hydrophobic cleft and polymorphic residues are shown, with colouring as described for panel A. C) Solvent-accessible surface representation of the ‘silent face’ of the *Pv*AMA1 model. The hydrophobic cleft and polymorphic residues are shown, with colouring as described for panel A.

**Figure 4:**
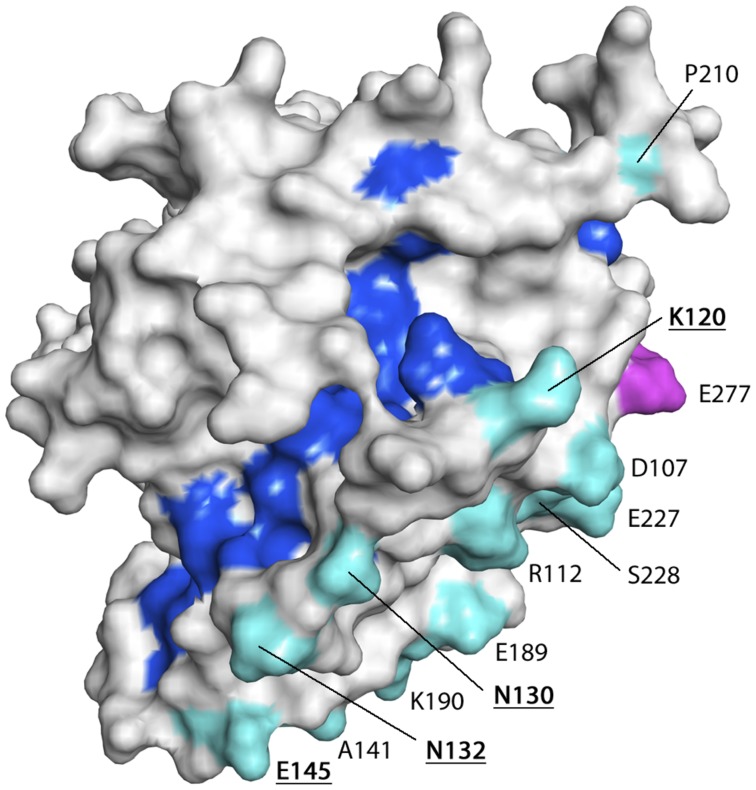
Proximity of the *Pv*AMA1 residues under selection to the hydrophobic ligand-binding cleft. Solvent-accessible surface representation of the *Pv*AMA1 model showing a top-view of the hydrophobic ligand-binding cleft. Binding cleft residues are highlighted in dark blue, DI polymorphic residues in cyan and a DII residue in magenta. Residues labeled with bold, underlined type are located in close proximity to the cleft and are predicted to be of functional importance.

All 22 polymorphic residues mapped to solvent-exposed surfaces of the *Pv*AMA1 molecule (Figure 3). Extreme bias in the distribution of polymorphisms was observed, with 21 of the 22 polymorphic residues located on one face of the *Pv*AMA1 structure (Figure 3B). Only the signal sequence polymorphic residue (Arg66) was located on the opposing face (Figure 3C). Four polymorphic residues (Lys120, Asn130, Asn132 and Glu145) were located proximal to the hydrophobic binding cleft in DI loops (Figure 4), three of which aligned with highly polymorphic *Pf*AMA1 residues associated with antigenic escape [38]. The *Pv*AMA1 Lys120 residue aligned with *Pf*AMA1 Tyr175 located within the c3 cluster; *Pv*AMA1 Asn132 and Glu145 aligned with *Pf*AMA1 Glu187 and His200, respectively, located within the c1 cluster (Figure 4, Figure S2).

### Population structure and clustering patterns of *Pv*AMA1 amino acid haplotypes

**Figure 5:**
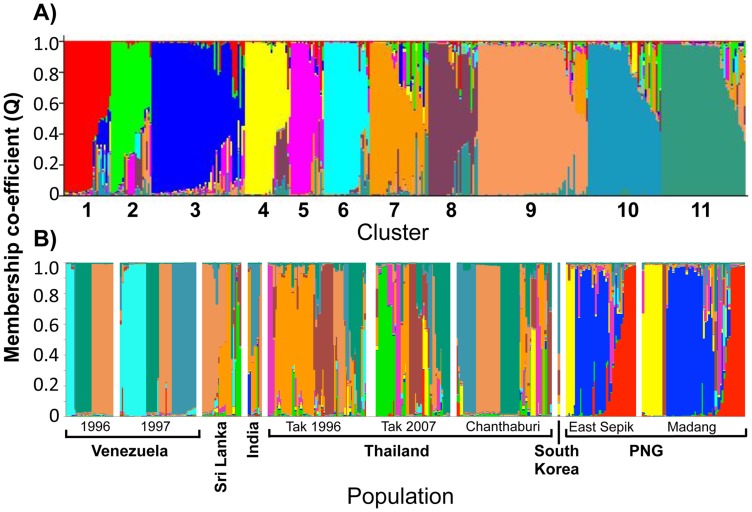
Clustering patterns of *Pv*AMA1 haplotypes. Structure analysis of *Pv*AMA1 haplotypes composed of 23 common amino acid polymorphisms. Haplotypes are shown according to A) the membership coefficient (Q) and B) geographic distribution of eleven clusters (K = 11).

To identify population structure and therefore genetically distinct subgroups among the 23-mer amino acid haplotypes, cluster analyses were performed. Analysis of (i) the LnP[D] curve, (ii) Δ*K* plot [63] and (iii) the distribution of clusters amongst haplotypes indicated that the haplotypes were optimally grouped into eleven clusters (*K* = 11; Figure 5A). Clusters were unevenly distributed among parasite populations with the majority of haplotypes in each geographic region having membership to only a few of the defined clusters (Figure 5B). This indicates substantial geographic differentiation, though only on a broad geographical scale as parasite populations from the same country showed similar clustering patterns (Figure 5B). The PNG haplotypes were classified into three clusters, which were found only in PNG, namely clusters 1 (15.6% of PNG haplotypes), 3 (33%) and 4 (15.6%). Thirty-three PNG haplotypes (32.3%) were admixed (i.e. <75% membership to any one cluster) (Figure 5B). Interestingly, all seven of the reference isolate haplotypes were admixed, suggesting that there may be additional clusters in other populations not yet surveyed (Figure S5).

**Figure 6:**
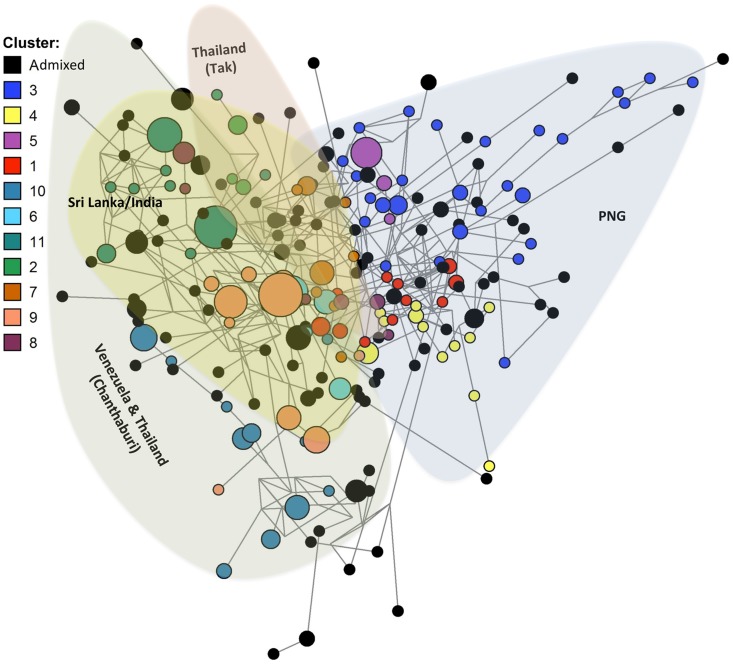
Network analysis of *Pv*AMA1 haplotypes. Haplotypes composed of 23 common amino acid polymorphisms were analysed using the Median Joining algorithm implemented in Phylogenetic Network version 4.6.1.1 software. Nodes represent the haplotypes and lines indicate connections between them. The size of each node indicates haplotype frequency. Colours indicated by the key depict the cluster membership as defined by Structure analyses.

The 210 unique 23-mer haplotypes formed a dense network with extremely complex relationships (Figure 6) however reducing the sample set to haplotypes with a frequency >1 revealed a more segmented network (Figure S6). Branching patterns in both networks correlated well with the cluster analyses described above and thus the country of origin, and ties between clusters were punctuated by admixed haplotypes (Figure 6, S6). A large number of admixed haplotypes were lost from the second network showing that these are mostly rare haplotypes and thus may be new recombinants (Figure S6). Interestingly, clusters from the South American and Asian populations overlapped, whereas all three clusters found in PNG were found in a distinct region of the network. This showed that PNG forms a distinct group of clusters more closely related to each other than clusters found in other parts of the world (Figure 6).

## Discussion

*Plasmodium vivax* is responsible for a large proportion of the global malaria burden, particularly in the Asia-Pacific region and thus must be targeted if malaria elimination is to be achieved. *P. vivax* is proving more difficult to eliminate than *P. falciparum* most likely due to its dormant liver stage, and there are fewer research tools available with which to study it [4], [68]. Furthermore, *P. vivax* is a more genetically diverse parasite than *P. falciparum* [69] suggesting a greater potential for vaccine escape. Vaccines could function as a key component of control and elimination campaigns, however the majority of current vaccine research is directed primarily toward *P. falciparum*. Research toward development of vaccines targeting *P. vivax* must therefore be prioritised. The aim of this study was to examine the global population structure of *Pv*AMA1, and thus to assess its feasibility as a vaccine candidate.

Consistent with published reports [11], [38], the genetic diversity of *Pv*AMA1 was extremely high, however there was substantial geographic variability with lower diversity in Venezuela compared to PNG and Thailand. As diversity increases with transmission intensity due to an increased effective population size and rates of recombination [70], it is likely that lower malaria transmission in Venezuela [71], [72] contributed to the lower genetic diversity observed. Higher genetic diversity in Asia is also consistent with the hypothesis that *P. vivax* originated in this region, with consequent founder effects and smaller effective population sizes in South America [73], [74].

Natural selection exerted by host immune responses, and recombination between genetically distinct clones during meiotic replication in the mosquito midgut are the two main mechanisms by which AMA1 genetic diversity is likely to be generated and maintained [30], [75]. Balancing selection acts to maintain key antigen polymorphisms at low to medium frequencies to enable the parasite to evade targeted immune responses [40]. Population genetic analyses have therefore been used previously to successfully identify important targets of host immune responses within the *P. falciparum* antigen genes, Merozoite Surface Protein 1 (MSP1) and AMA1 [39], [40]. The majority of the diversity and balancing selection in *Pv*AMA1 mapped to DI, as has been observed previously for both *P. falciparum* and *P. vivax* [29], [31], [76]. Although *P*vAMA1 DII has been reported as highly immunogenic [22], [23] and there is some evidence of balancing selection in DII [75], our results are consistent with those of other studies that suggest DI is the dominant target of naturally acquired host immune responses [29], [35], [77]. Negative values of Tajima's D obtained by Taylor et al., [78] following analysis of mitochondrial (mtDNA) sequences from PNG provide further evidence that the positive values of Tajima's D obtained for *Pvama1* DI are the result of balancing selection acting on this region. Domain I therefore represents the strongest candidate domain for incorporation into a potential *Pv*AMA1 vaccine.

As reported previously [77], extreme bias in the distribution of the polymorphic residues under selection was observed. Twenty-one of the 22 ‘common’ residues were located on one side of the *Pv*AMA1 ectodomain, suggesting that this ‘face’ is potentially more exposed and accessible to host immune responses, consistent with our findings that these residues are under strong balancing selection. Only two *Pv*AMA1 polymorphisms mapped to the *Pf*AMA1 c1L cluster of polymorphisms (positions 196 to 207 of DI), which is most strongly associated with antibody escape [38]. A total of nine *Pv*AMA1 polymorphisms mapped to the four additional clusters of *Pf*AMA1 residues also associated with immune evasion [38]. The highly polymorphic residues of the *Pf*AMA1 c1L cluster are located on loops, which are thought to protect a functionally critical binding trough by forming a ‘shield’ to divert host antibody responses [12]. Differences in the positioning of *Pf*AMA1 and *Pv*AMA1 polymorphic residues suggests differential organisation and presentation of the domain I loops. Four of the 23 ‘common’ polymorphisms identified (E145A, P210S, K352E and R438H) did however map to predicted *Pv*AMA1 B-cell epitopes [79]. Consistent with the findings of Zakeri et al., [79], residue E145A was identified in the present study as one of four ‘common’ polymorphic residues located proximal to the *Pv*AMA1 ligand-binding cleft, suggesting a potential role for this residue in immune evasion. Additionally, two ‘common’ polymorphisms (Q380K and L384P/R) were reported to cause changes in protein polarity and hydrophilicity and therefore may alter protein structure [79]. Dias *et al*., (2011) also reported decreased epitope binding scores or loss of the predicted linear B cell epitope due to these two polymorphisms, either together or in combination with proximal polymorphisms [80]. It will now be important to determine the functional relevance of the 23 ‘common’ polymorphic amino acids identified in this survey of global *Pv*AMA1 diversity.

An effective *Pv*AMA1 vaccine must include alleles (haplotypes) that induce host immune responses that are sufficiently broad to cover the existing antigenic diversity [36], [70]. Neafsey *et al*., (2012) recently reported that generating broad cross-reactive immune responses against highly polymorphic antigens, such as AMA1, may present a far greater challenge for *P. vivax* than for *P. falciparum* on account of higher *P. vivax* sequence diversity [69]. Our observation that only 15% of *Pv*AMA1 haplotypes were shared between populations and the complex network connected by rare admixed haplotypes suggest that covering diversity will be challenging. Although highly diverse, cluster analysis of the 210 *Pv*AMA1 minimal haplotypes identified 11 distinct subgroups. The number of clusters identified was almost double that identified amongst global *Pf*AMA1 sequences ([81], [82], n = 6), however deep sampling of an African population suggested up to 16 *Pf*AMA1 clusters [38]. In stark contrast to the relatively even global distribution of known *Pf*AMA1 diversity [81], [82], substantial geographical differentiation between populations was observed for *Pv*AMA1. The observation that the majority of PNG *Pv*AMA1 sequences were grouped into three closely related clusters not found outside PNG indicates that PNG harbours distinct *Pv*AMA1 sequences with negligible overlap between Asian and South American populations, and strengthens previous findings that the PNG *P. vivax* population is distinct from other worldwide populations [78], [83].

The burden of *P.vivax* in PNG is amongst the highest in the world [84], however *P. vivax* diversity, including that associated with antigens such as AMA1, has been rarely studied in PNG. Few haplotypes were shared between the two PNG populations. However, the PNG sequences and haplotypes did not bifurcate by province in the phylogenetic, cluster or network analyses. Although sympatric *P. falciparum* populations in the same two regions of PNG are genetically distinct [43], the *Pv*AMA1 clustering patterns in the two *P. vivax* populations of PNG were almost identical suggesting mixing of *Pv*AMA1 alleles between the two populations. However, balancing selection is also likely to influence these patterns [39]. The lack of similarities to other worldwide populations may be the result of the geographical isolation and large effective population size of PNG parasites, or another variable such as a specific host genetic adaptation and thus warrants further investigation. Additionally, based on more slowly evolving mtDNA haplotypes, that the PNG *P. vivax* population has higher genetic diversity and is differentiated from other populations worldwide may be a result of founder effects combined with a long history of intense *P. vivax* transmission in PNG [78], [83], [85]. A further explanation might be adaptation to the range of unique red blood cell polymorphisms in the PNG human population, including one that is associated with protection against infection with *P. vivax* [86]. The fact the *P. vivax* population in PNG is genetically distinct compared to other global parasite populations suggests that a *Pv*AMA1-based malaria vaccine effective in other parts of the world may not be as successful in PNG.

In order to determine if development of a globally effective *Pv*AMA1 vaccine is feasible, it will be important to establish whether *Pv*AMA1 haplotypes from different clusters are antigenically distinct, as has been previously observed for *Pf*AMA1 [81]. Indeed, it was recently reported that *Pf*AMA1 sequence differences may not necessarily be strong predictors of antigenic differences or the level of cross-inhibitory antibody activity because not all polymorphic residues contribute equally to antibody binding and escape [87]. Whether the antigenic diversity of inhibitory antibody epitopes is similarly limited for *Pv*AMA1 remains unknown. The functional contribution of each of the polymorphisms identified in this study to inhibitory antibody activity must now be determined, so that the number of alleles required to cover the breadth of global *Pv*AMA1 diversity can be established.

As *P. vivax* cannot be continuously maintained in culture, recombinant proteins used for immunoepidemiological studies and vaccine development are typically derived from the reference strain, *Sal-1*. Inclusion of alleles that are not representative of natural parasite populations within a malaria vaccine may result in poor vaccine efficacy or selection for variants not included in the vaccine, and waning of vaccine efficacy over time [38]. It was therefore an important finding of this study that the reference strain *Sal-1 Pv*AMA1 haplotype was not found in the global dataset. Historically, despite widespread knowledge of high diversity and strain-specific immunity in malaria vaccine candidates, the naturally circulating genetic diversity has rarely been considered when developing candidate vaccines [82] and this may explain the poor clinical efficacy observed [88], [89]. The identical *Belem* and *Palo Alto* haplotypes and the *Chesson I* haplotype were observed but were restricted to one Thai population. Alleles of the commonly used reference isolates for *P. vivax* vaccine design do not circulate widely, if at all, amongst natural populations and this may have serious implications for development of a *Pv*AMA1 vaccine based on these strains.

In summary, we have demonstrated that the global genetic diversity of *Pv*AMA1 is exceptionally high and geographically structured, and that domain I is a dominant target of balancing selection. Analyses of haplotypes based only on common amino acid polymorphisms predicted to contribute to antigenic diversity demonstrated that an enormous amount of diversity must be considered in developing a broadly efficacious *Pv*AMA1 vaccine. Importantly however, the cluster and network analyses provide a framework upon which to select a panel of broadly representative haplotypes for inclusion in a future *Pv*AMA1 vaccine. Functional testing must now be performed to investigate the contribution of the polymorphic residues identified to antibody binding and escape, and also to determine which combination of haplotypes might induce antibodies capable of providing broad coverage against the global antigenic diversity of *Pv*AMA1.

## Supporting Information

Figure S1 **Phylogenetic analysis of PNG** ***Pvama1*** **sequences.** Neighbor-Joining tree constructed using 102 unique *Pvama1* ectodomain sequences from PNG. Circles indicate sequences from Madang and triangles, East Sepik. Circle/triangle colours correspond to the cluster membership (Figure 5) of each sequence: cluster 1 (red), cluster 3 (blue), cluster 4 (yellow), cluster 5 (pink), cluster 8 (maroon) and admixed (black). The *Sal-1* reference sequence was also included as indicated. The tree was constructed using 10,000 bootstrap replicates, with only values >70% shown. The tree is drawn to scale, with branch lengths in the same units as the evolutionary distance (number of differences) used to infer the tree. All ambiguous positions were removed for each sequence pair.
(PDF)

Figure S2 **Alignment of** ***P. falciparum*** **and** ***P. vivax*** **AMA1 protein sequences.** *P. vivax Sal-1* (GenBank: AF063138) and *P. falciparum* 3D7 (XM_001347979) were aligned using MEGA version 5.0 [51]. Numbers indicate the position of residues relative to those of the *P. falciparum* sequence. Gaps are indicated by dashes. Red bold type indicates the 23 common *P. vivax* amino acids predicted to be immunologically relevant. Black bold type indicates residues that are polymorphic in both species [77]. The domain boundaries are demarcated by vertical lines, as indicated. Boxes indicate the positions of antigenic *P. falciparum* amino acid clusters, c1-3 [66]; Grey shading indicates antigenic escape residues in c1L [38].
(PDF)

Figure S3 **Natural selection within** ***Pvama1*** **for isolates from Venezuela and Thailand.** Sliding window analysis of Tajima's D was performed for the Venezuelan 1997 population (i), the two Thai Tak province populations (ii; the solid line represents the Tak 1996 population and the dashed line represents the Tak 2007 population) and the Thai Chanthaburi population (iii). A window size of 100 and a step size of 3 were used. Horizontal dashed lines indicate the significance threshold (p = 0.05); a single asterisk indicates values for which p<0.05.
(PDF)

Figure S4 **Worldwide distribution of** ***Pv*** **AMA1 40-mer haplotypes.** Based on the analysis of the 40 NS amino acid polymorphism haplotypes, pie charts depicting the relative frequencies of the 219 haplotypes identified were drawn for each parasite population. Coloured segments indicate haplotypes that are present in more than one population; grey indicates haplotypes present in only one population. Only one haplotype was identical to reference strains (*Belem/Palo Alto*), therefore haplotypes from the remaining reference strains are shown in grey. Sample size and origin are indicated.
(PDF)

Figure S5 **Cluster membership of the** ***P. vivax*** **reference strains.** Haplotypes analysed using the program Structure [61], [62] were found to be optimally distributed among eleven clusters (*K* = 11). Colours indicate the proportion of each reference strain (membership coefficient, Q) belonging to each of the different clusters identified.
(PDF)

Figure S6 **Network analysis of** ***Pv*** **AMA1 haplotypes with a frequency >1.** Haplotypes composed of 23 common amino acid polymorphisms with a frequency >1 were analysed using the Median Joining algorithm implemented in Phylogenetic Network version 4.6.1.1 software. Coloured nodes represent the haplotypes and lines indicate connections between them. The size of each node indicates haplotype frequency.
(PDF)

Table S1 **Published** ***Pvama1*** **sequences obtained from GenBank.**
(DOC)

Table S2 **Summary of** ***Pv*** **AMA1 polymorphisms.** All 40-mer haplotypes for the 372 global sequences are summarised here. The polymorphic amino acid residue, and corresponding nucleotide polymorphism(s) are listed, as are the reference *Sal-1* sequence and variant codons for each polymorphic site. Sites identical to the *Sal-1* sequence are indicated by a dot. The number of sequences identical to *Sal-1* at each polymorphic site are also listed. The 23 common polymorphisms are shown in bold.
(XLSX)
