# Supplementary figures and images for: Global Population Structure of the Genes Encoding the Malaria Vaccine Candidate, Plasmodium vivax Apical Membrane Antigen 1 (PvAMA1)
Source: PLoS Negl Trop Dis. 2013 Oct 31;7(10):e2506. doi: 10.1371/journal.pntd.0002506 (PMC3814406; doi:10.1371/journal.pntd.0002506)

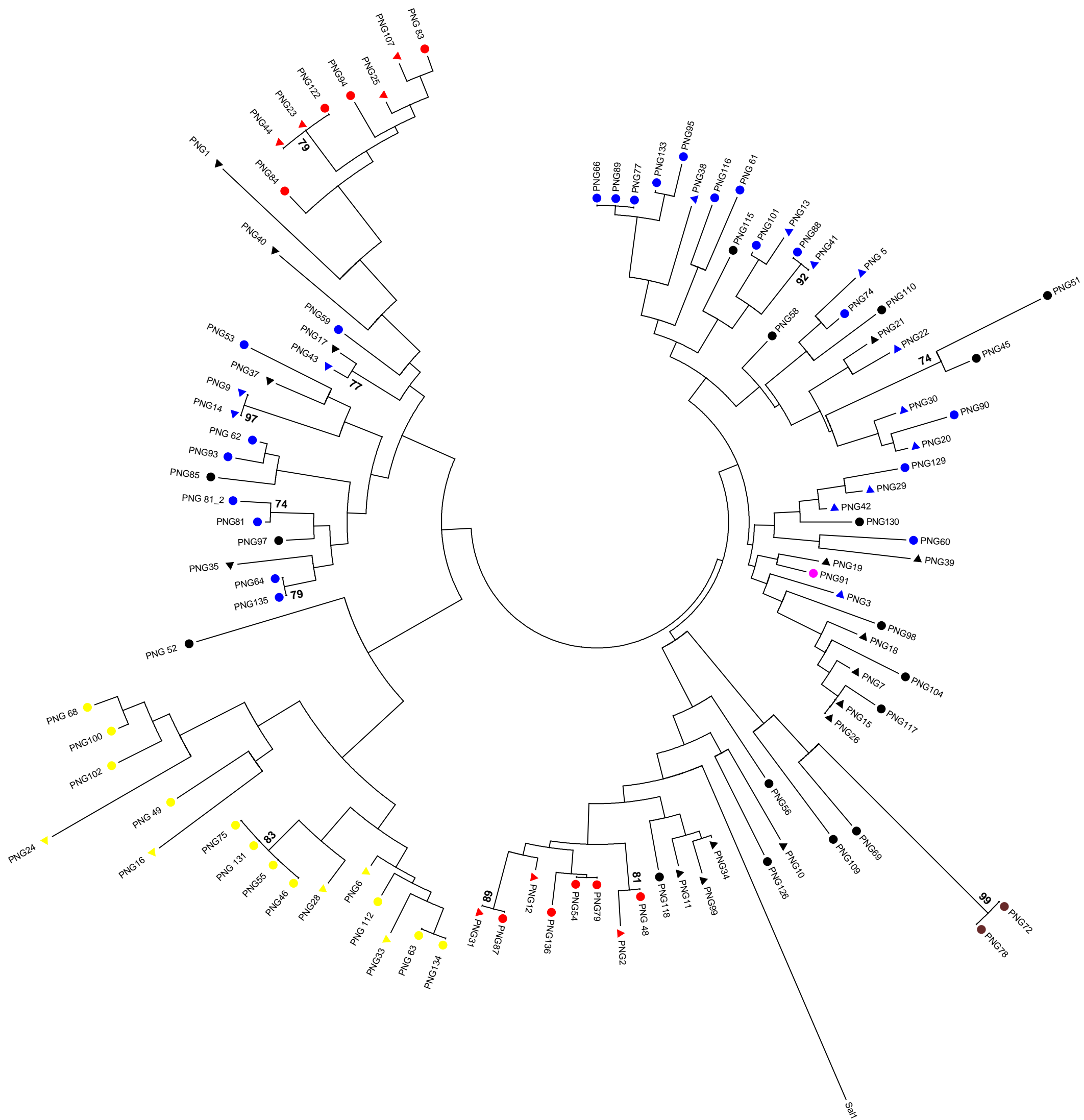

Supplement: Figure S1 — Phylogenetic analysis of PNG Pvama1 sequences. Neighbor-Joining tree constructed using 102 unique Pvama1 ectodomain sequences from PNG. Circles indicate sequences from Madang and triangles, East Sepik. Circle/triangle colours correspond to the cluster membership (Figure 5) of each sequence: cluster 1 (red), cluster 3 (blue), cluster 4 (yellow), cluster 5 (pink), cluster 8 (maroon) and admixed (black). The Sal-1 reference sequence was also included as indicated. The tree was constructed using 10,000 bootstrap replicates, with only values >70% shown. The tree is drawn to scale, with branch lengths in the same units as the evolutionary distance (number of differences) used to infer the tree. All ambiguous positions were removed for each sequence pair. (PDF) [file pntd.0002506.s001.pdf]

i) Venezuela

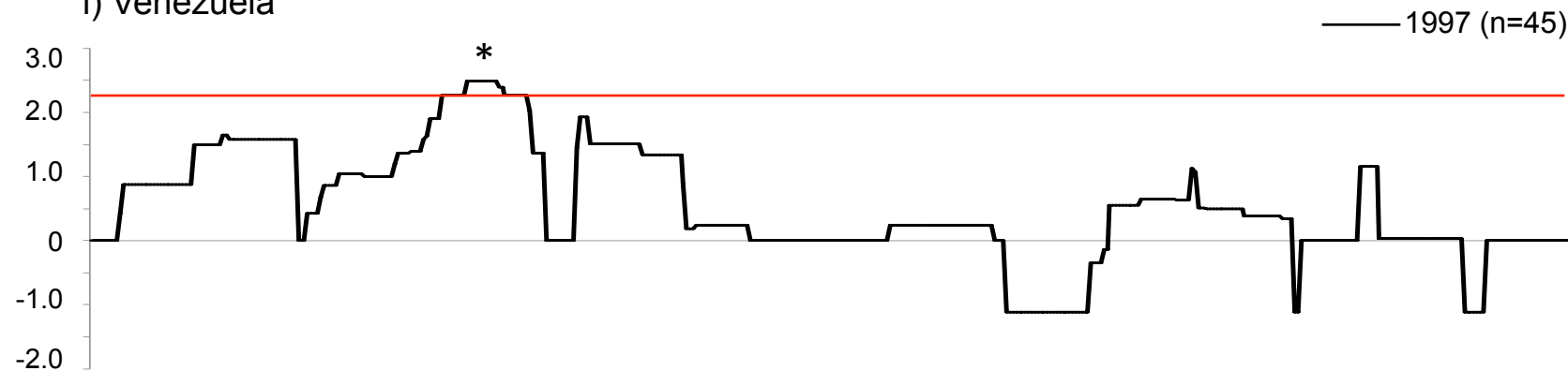

ii) Thailand

— Tak province 1996 (n=58)    ..... Tak province 2007 (n=44)

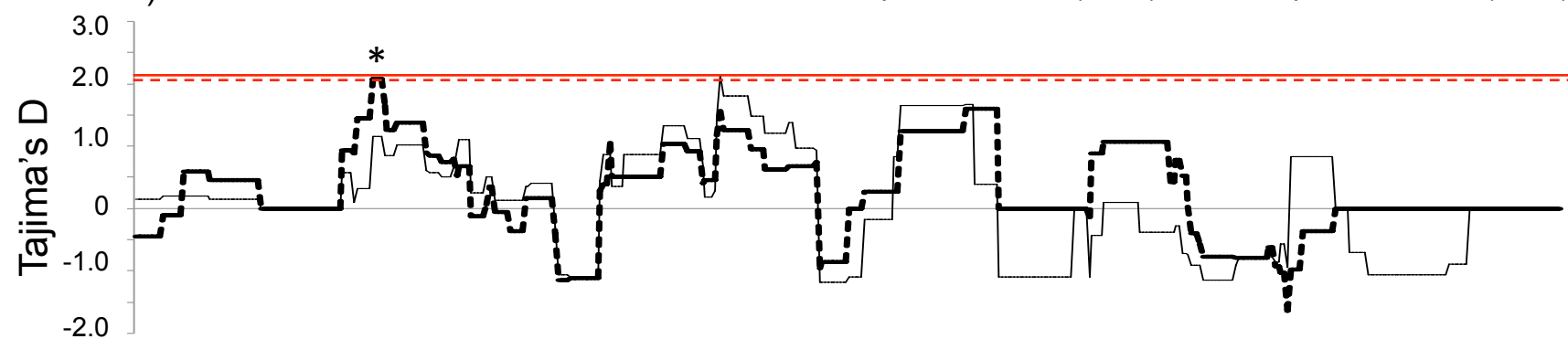

iii) Thailand

Chanthaburi province (n=56)

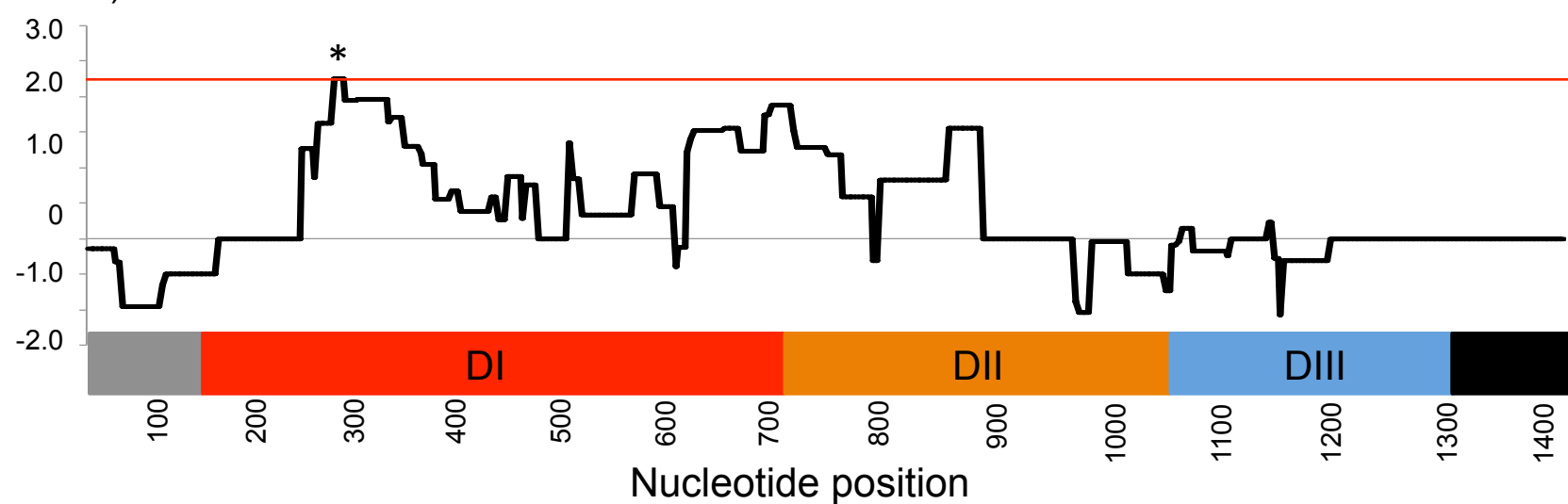

Supplement: Figure S3 — Natural selection within Pvama1 for isolates from Venezuela and Thailand. Sliding window analysis of Tajima's D was performed for the Venezuelan 1997 population (i), the two Thai Tak province populations (ii; the solid line represents the Tak 1996 population and the dashed line represents the Tak 2007 population) and the Thai Chanthaburi population (iii). A window size of 100 and a step size of 3 were used. Horizontal dashed lines indicate the significance threshold (p = 0.05); a single asterisk indicates values for which p<0.05. (PDF) [file pntd.0002506.s003.pdf]

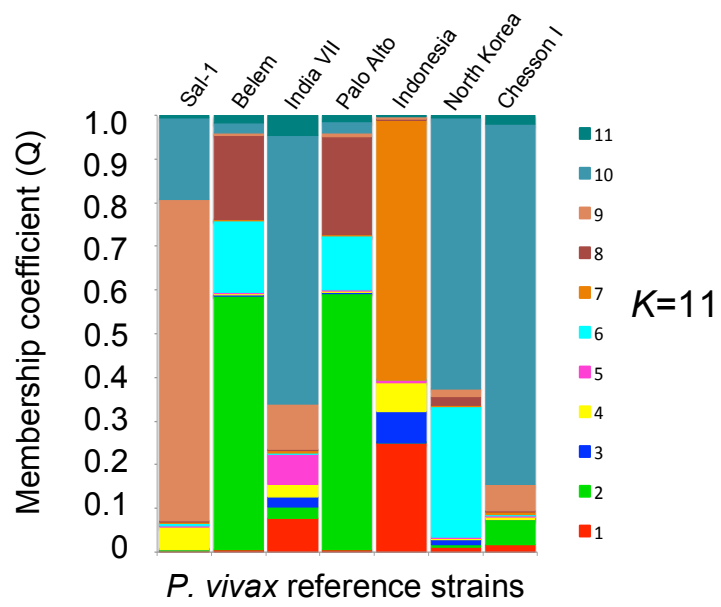

Supplement: Figure S5 — Cluster membership of the P. vivax reference strains. Haplotypes analysed using the program Structure [61], [62] were found to be optimally distributed among eleven clusters (K = 11). Colours indicate the proportion of each reference strain (membership coefficient, Q) belonging to each of the different clusters identified. (PDF) [file pntd.0002506.s005.pdf]

# Cluster:

Admixed

3

4

5

1

10

6

11

2

7

9

8

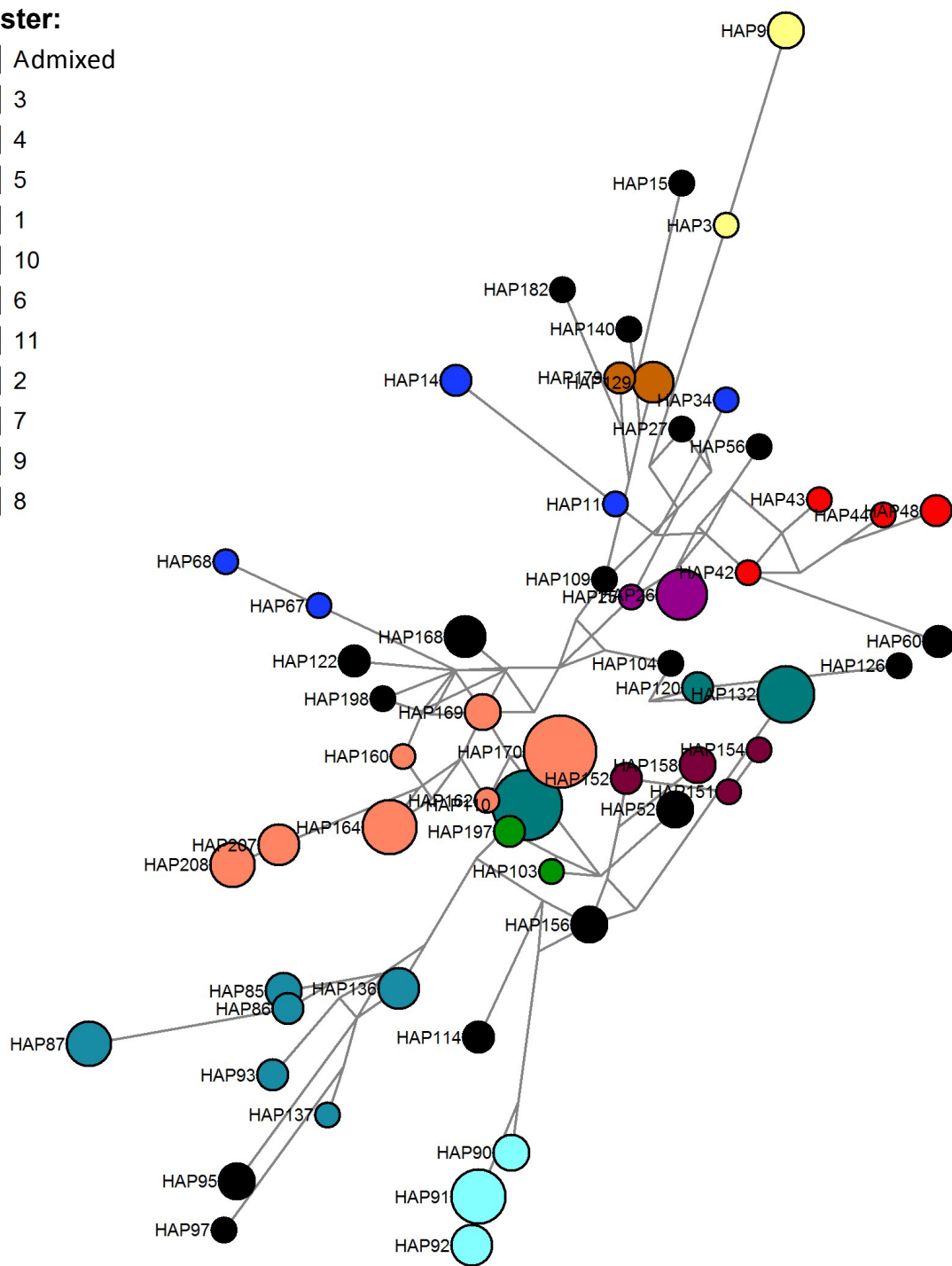

Supplement: Figure S6 — Network analysis of Pv AMA1 haplotypes with a frequency >1. Haplotypes composed of 23 common amino acid polymorphisms with a frequency >1 were analysed using the Median Joining algorithm implemented in Phylogenetic Network version 4.6.1.1 software. Coloured nodes represent the haplotypes and lines indicate connections between them. The size of each node indicates haplotype frequency. (PDF) [file pntd.0002506.s006.pdf]
