# Supplementary material for: Global Population Structure of the Genes Encoding the Malaria Vaccine Candidate, Plasmodium vivax Apical Membrane Antigen 1 (PvAMA1)
Source: PLoS Negl Trop Dis. 2013 Oct 31;7(10):e2506. doi: 10.1371/journal.pntd.0002506 (PMC3814406; doi:10.1371/journal.pntd.0002506)
Supplement: Figure S2 — Alignment of P. falciparum and P. vivax AMA1 protein sequences. P. vivax Sal-1 (GenBank: AF063138) and P. falciparum 3D7 (XM_001347979) were aligned using MEGA version 5.0 [51]. Numbers indicate the position of residues relative to those of the P. falciparum sequence. Gaps are indicated by dashes. Red bold type indicates the 23 common P. vivax amino acids predicted to be immunologically relevant. Black bold type indicates residues that are polymorphic in both species [77]. The domain boundaries are demarcated by vertical lines, as indicated. Boxes indicate the positions of antigenic P. falciparum amino acid clusters, c1-3 [66]; Grey shading indicates antigenic escape residues in c1L [38]. (PDF) [file pntd.0002506.s002.pdf]

```
                                                                                                   DI
                          5          15         25         35         45         55         65         75
P. falciparum (3D7)  MRKLYCVLLL SAFEFTYMIN FGRGQNYWEH PYQNSDVYRP INEHREHPKE YEYPLHQEHT YQQEDSGEDE NTLQHAYPID
P. vivax (Sal-1)     MNKIYYIIFL SAQCLVHIGK CGRNQKPSRL TRSANNVLLE KGP------- ---------- ---------- ----------

                          85         95         105        115        125        135        145        155
P. falciparum (3D7)  HEGAEPAPQE QNLFSSIEIV ERSNYMGNPW TEYMAKYDIE EVHGSGIRVD LGEDAEVAGT QYRLPSGKCP VFGKGIIIEN
P. vivax (Sal-1)     ---------- --------TV ERSTRMSNPW KAFMEKYDIE RTHSSGVRVD LGEDAEVENA KYRIPAGRCP VFGKGIVIEN

                          165        175        185        195        205        215        225        235
P. falciparum (3D7)  SNTTFLTPVA TGNQYLKDGG FAFPPTEPLM SPMTLDEMRH FYKDNKYVKN LDELTLCSRH AGNMIPDNDK NSNYKYPAVY
P. vivax (Sal-1)     SDVSFLRPVA TGDQKLKDGG FAFPNANDHI SPMTLANLKE RYKDNVEMMK LNDIALCRTH AASFVMAGDQ NSSYRHPAVY
                                 c3                    c1                                              c1          DII

                          245        255        265        275        285        295        305        315
P. falciparum (3D7)  DDKDKKCHIL YIAAQENNGP RYCNKDESKR NSMFCFRPAK DISFQNYTYL SKNVVDNWEK VCPRKNLQNA KFGLWVDGNC
P. vivax (Sal-1)     DEKEKTCHML YLSAQENMGP RYCSPDAQNR DAVFCFKPDK NESFENLVYL SKNVRNDWDK KCPRKNLGNA KFGLWVDGNC
                       c2                             c3 c3                   c2

                          325        335        345        355        365        375        385        395
P. falciparum (3D7)  EDIPHVNEFP AIDLFECNKL VFELSASDQP KQYEQHLTDY EKIKEGFKNK NASMIKSAFL PTGAFKADRY KSHGKGYNWG
P. vivax (Sal-1)     EEIPYVKEVE AEDLRECNRI VFGASASDQP TQYEEEMTDY QKIQQGFRQN NREMIKSAFL PVGAFNSDNF KSKGRGFNWA

                                                                            DIII
                          405        415        425        435        445        455        465        475
P. falciparum (3D7)  NYNTETQKCE IFNVKPTCLI NNSSYIATTA LSHPIEVENN FPCSLYKDEI MKEIERESKR IKLNDNDDEG NKKIIAPRIF
P. vivax (Sal-1)     NFDSVKKKCY IFNTKPTCLI NDKNFIATTA LSHPQEVDLE FPCSIYKDEI EREIKKQSRN MNLYSVDGER ---IVLPRIF

                          485        495        505        515        525        535        545        555
P. falciparum (3D7)  ISDDKDSLKC PCDPEMVSNS TCRFFVCKCV ERRAEVTSNN EVVVKEEYKD EYADIPEHKP TYDKMKIIIA SSAAVAVLAT
P. vivax (Sal-1)     ISNDKESIKC PCEPERISNS TCNFYVCNCV EKRAEIKENN QVVIKEEFRD YYEN-GEEK- SNKQMLLIII GITGGVCVVA

                          565        575        585        595        603
P. falciparum (3D7)  ILMVYLYKRK GNAEKYDKMD EPQDYGKSNS RNDEMLDPEA SFW
P. vivax (Sal-1)     LASMAYFRKK ANNDKYDKMD QAEGYGKPTT RKDEMLDPEA SFW
```
