# Supplementary material for: Global Population Structure of the Genes Encoding the Malaria Vaccine Candidate, Plasmodium vivax Apical Membrane Antigen 1 (PvAMA1)
Source: PLoS Negl Trop Dis. 2013 Oct 31;7(10):e2506. doi: 10.1371/journal.pntd.0002506 (PMC3814406; doi:10.1371/journal.pntd.0002506)
Supplement: Figure S4 — Worldwide distribution of Pv AMA1 40-mer haplotypes. Based on the analysis of the 40 NS amino acid polymorphism haplotypes, pie charts depicting the relative frequencies of the 219 haplotypes identified were drawn for each parasite population. Coloured segments indicate haplotypes that are present in more than one population; grey indicates haplotypes present in only one population. Only one haplotype was identical to reference strains (Belem/Palo Alto), therefore haplotypes from the remaining reference strains are shown in grey. Sample size and origin are indicated. (PDF) [file pntd.0002506.s004.pdf]

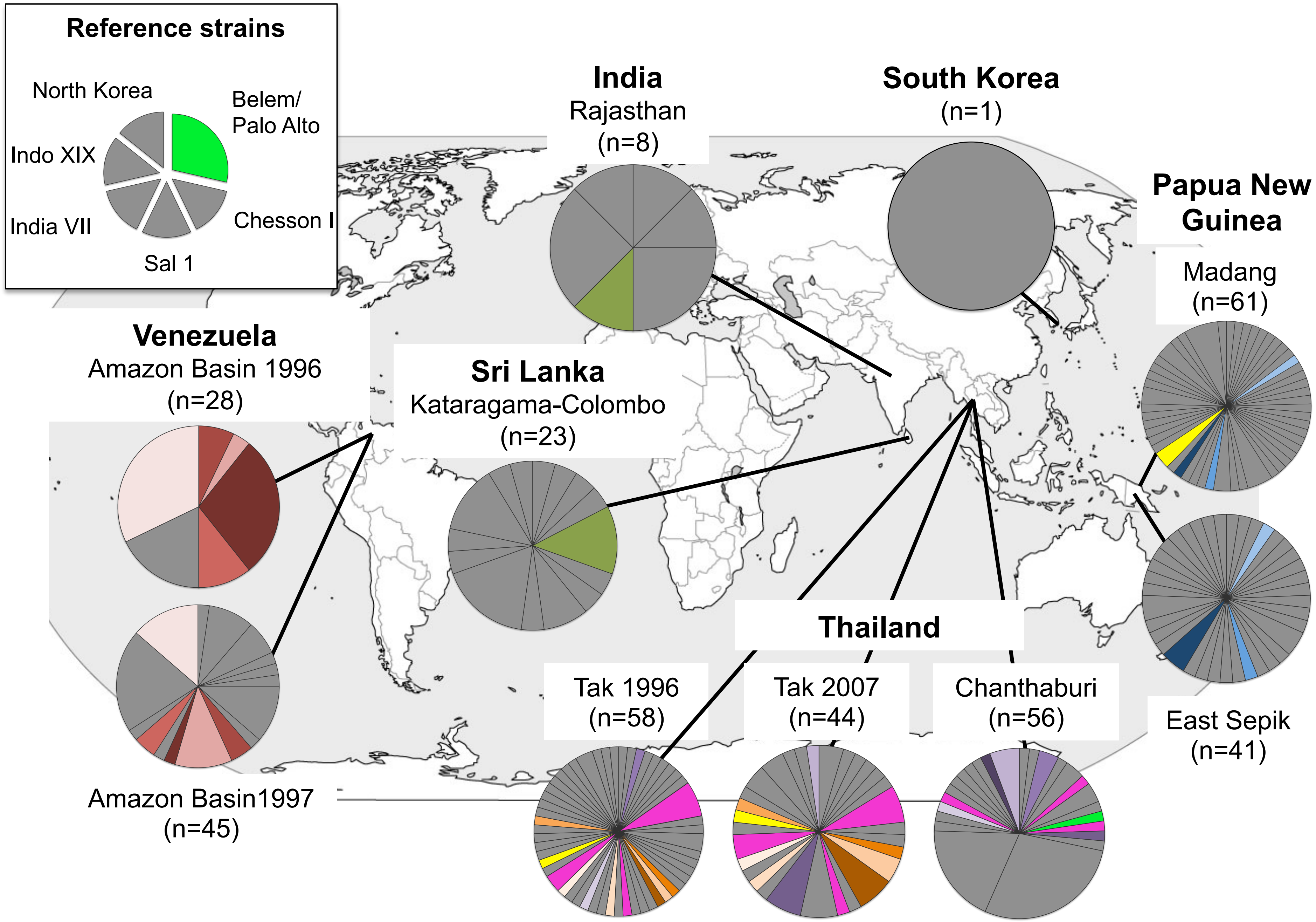
Reference strains
North Korea
Belem/
Palo Alto
Indo XIX
India VII
Chesson I
Sal 1
India
Rajasthan
(n=8)
South Korea
(n=1)
Papua New
Guinea
Madang
(n=61)
Venezuela
Amazon Basin 1996
(n=28)
Sri Lanka
Kataragama-Colombo
(n=23)
Thailand
Tak 1996
(n=58)
Tak 2007
(n=44)
Chanthaburi
(n=56)
East Sepik
(n=41)
Amazon Basin1997
(n=45)
