# Supplementary material for: Global Population Structure of the Genes Encoding the Malaria Vaccine Candidate, Plasmodium vivax Apical Membrane Antigen 1 (PvAMA1)
Source: PLoS Negl Trop Dis. 2013 Oct 31;7(10):e2506. doi: 10.1371/journal.pntd.0002506 (PMC3814406; doi:10.1371/journal.pntd.0002506)
Supplement: Table S1 — Published Pvama1 sequences obtained from GenBank. (DOC) [file pntd.0002506.s007.doc]

**Supplementary Table 1: Published *Pvama1* sequences obtained from GenBank**

| **Region** | **Country** | **Location** | **Year of collection** | **Disease classification** | **GenBank accession numbers** | **Reference** |
| --- | --- | --- | --- | --- | --- | --- |
| South America | Venezuela | Padamo River, Amazon Basin | 1996,1997 | Asymptomatic and symptomatic | EU346015-EU346087 | Ord *et al*., (2008)^1^ |
|  |  |  |  |  |  |  |
|  | Sri Lanka | Kataragama & Colombo | Not stated | Symptomatic | EF218679–EF218701 | Gunasekera *et al*., (2007)^2^ |
|  |  |  |  |  |  |  |
|  | India | Bikaner region of Rajasthan | Not stated | Symptomatic | EF025187–EF025197 | Rajesh *et al*., (2007)^3^ |
|  |  |  |  |  |  |  |
| Asia |  | Tak Province (bordering Myanmar) | 1996, 2007 | Symptomatic | FJ784891- | Putaporntip *et a*l., |
|  | Thailand | Chantaburi Province (bordering Cambodia) | 2007 | Symptomatic | FJ785121 | (2009)^4^ |
|  | South Korea | Not stated | Not stated | Not stated | GU476488 | GenBank |
| **Reference strains** |  |  | | |  |  |
| Salvador-1 | El Salvador | Sal-1 | | | AF063138 | Collins *et* al., (1972)^5^ |
|  |  |  | | |  |  |
| Primate-adapted strains | New Guinea, Brazilian Amazon, India, Indonesia, Vietnam, North Korea | Chesson I; Belem strain; India VII; Indonesia XIX; Palo Alto; North Korea | | | EU395587-EU395593 | Ntumngia *et al*., (2009)^6^ |

^1^ Ord RL, Tami A, Sutherland CJ (2008) ama1 genes of sympatric Plasmodium vivax and P. falciparum from Venezuela differ significantly in genetic diversity and recombination frequency. PLoS One 3: e3366

^2^ Gunasekera AM, Wickramarachchi T, Neafsey DE, Ganguli I, Perera L, et al. (2007) Genetic diversity and selection at the Plasmodium vivax apical membrane antigen-1 (PvAMA-1) locus in a Sri Lankan population. Mol Biol Evol 24: 939-947

^3^ Rajesh V, Elamaran M, Vidya S, Gowrishankar M, Kochar D, et al. (2007) Plasmodium vivax: genetic diversity of the apical membrane antigen-1 (AMA-1) in isolates from India. Exp Parasitol 116: 252-256

^4^ Putaporntip C, Jongwutiwes S, Grynberg P, Cui L, Hughes AL (2009) Nucleotide sequence polymorphism at the apical membrane antigen-1 locus reveals population history of Plasmodium vivax in Thailand. Infect Genet Evol 9: 1295-1300

^5^ Collins WE, Contacos PG, Krotoski WA, Howard WA (1972) Transmission of four Central American strains of Plasmodium vivax from monkey to man. J Parasitol 58: 332-335

^6^ Ntumngia FB, McHenry AM, Barnwell JW, Cole-Tobian J, King CL, et al. (2009) Genetic variation among Plasmodium vivax isolates adapted to non-human primates and the implication for vaccine development. Am J Trop Med Hyg 80: 218-227
